# Trajectory of depressive symptoms over adolescence in autistic and neurotypical youth

**DOI:** 10.1186/s13229-024-00600-w

**Published:** 2024-05-02

**Authors:** Blythe A. Corbett, Rachael A. Muscatello, Trey McGonigle, Simon Vandekar, Christina Burroughs, Sloane Sparks

**Affiliations:** 1 Department of Psychiatry and Behavioral Sciences, Vanderbilt University Medical Center, 1500 21st Avenue South, Nashville, TN 37212 USA; 2 Vanderbilt Kennedy Center, Vanderbilt University Medical Center, Nashville, TN USA; 3 Department of Psychology, Vanderbilt University, Nashville, TN USA; 4 Department of Biostatistics, Vanderbilt University Medical Center, Nashville, TN USA

**Keywords:** Autism, Puberty, Adolescence, Depression, Development

## Abstract

**Background:**

Adolescence coincides with a dramatic rise in the onset of psychiatric conditions including depression. Depression symptoms may be particularly prevalent and impairing for youth with autism spectrum disorder (ASD). While prior research suggests adolescence is associated with worsening depression symptoms for typically developing (TD) and autistic youth, it is unclear if they follow a similar course.

**Method:**

The study examined the trajectory of depressive symptoms in autistic and neurotypical youth over a 4-year longitudinal study using linear and logistic mixed effects models. In youth with clinically relevant depressive scores (t-score > 65), moderating factors (i.e., diagnosis, age, puberty, sex) were explored. During Year 1, the sample included 244 youth 10-to-13 years: 140 in the ASD group (36 females) and 104 in the TD group (46 females).

**Results:**

Autistic youth had elevated depression scores compared to TD peers (*p* < 0.001) and females were higher than males in both groups (*p* = 0.001). There was significant diagnosis by age (*p* < 0.001) and diagnosis by pubertal stage (*p* < 0.05) interactions. In the ASD group, elevated depressive scores presented in early adolescence and decreased during middle adolescence and puberty, whereas the TD group showed the opposite trend with an increase in depression symptoms with advancing development.

**Limitations:**

Limitations include an unequal sex distribution (fewer females), non-representative autistic sample (e.g., cognition and race/ethnicity), and potential confound of the COVID-19 pandemic.

**Conclusions:**

Autistic youth present with higher rates of depressive symptoms early in development; yet, approaching middle adolescence and puberty, the symptom trajectory in the autistic youth declines coinciding with an increase in the TD youth. While group trajectories are divergent, they lead to similar levels of depression in late adolescence with higher symptoms in females. Findings suggest a period of quiescence in depressive symptomology influenced by biopsychosocial factors impacting affective profiles.

**Supplementary Information:**

The online version contains supplementary material available at 10.1186/s13229-024-00600-w.

## Background

Adolescence marks the developmental transition of juvenile social and cognitive processes to adult levels of functioning and independence. It is a dynamic time to negotiate amidst many psychological, social, and physiological changes [1]. Adolescence is generally divided into three age ranges—*early* (10–14 years), *middle* (15–17 years) and *late* adolescence/young adulthood (17–24). Not surprisingly, adolescence is a period impacted by a dramatic rise in the onset of psychiatric conditions such that half of those diagnosed with mental illness during their lifetime, will have their onset by 14 years of age [2].

Depression is a common albeit debilitating mental health condition characterized by feelings of sadness, hopelessness and diminished interest or pleasure in activities for which one previously enjoyed [3]. To receive a diagnosis of depression, the clinically significant distress must persist for at least two weeks and impact important areas of functioning (e.g., social, academic, occupational). Based on the in-person National Comorbidity Survey Adolescent Supplement conducted in 10,123 adolescents aged 13–18 years, the estimated lifetime prevalence of depression in the United States is 17.5% for adolescents [4]. However, even if a full diagnosis is not met, the subclinical sequelae from depressive symptoms can have a significant impact on emotional, cognitive, social, and behavioral functioning. Depression symptoms may be particularly prevalent and impairing for youth with autism spectrum disorder (ASD), as up to 26% of autistic youth experience depression symptoms [5, 6]. While prior research suggests the transition from childhood to adolescence is associated with worsening depression symptoms for both typically developing (TD) [7, 8] and autistic youth [5, 6], it is unclear if depression symptoms in youth with ASD follow a similar trajectory to TD youth.

### Autism spectrum disorder

Autism spectrum disorder (ASD) is a neurodevelopmental disorder differentiated by significant challenges in reciprocal social communication and patterns of narrow, stereotyped, or repetitive interests and behaviors [3].^1^ Throughout the document, the term “ASD” is used to reference individuals with a confirmed DSM-5 [3] diagnosis of autism. Historically, a male bias has been reported with an average 4:1 male-to-female ratio [9]. In recent years, evidence has emerged suggesting that the male-to-female ratio may be closer to 2:1 or 3:1 with a distinct female phenotype, which may lead to under-diagnosis in females [10, 11].

An important aspect of ASD is that individuals often experience poor adaptation to novelty or change including developmental transitions [3, 12]. As such, adolescence has been proposed as a challenging developmental period [13]. Some research has shown that psychosocial challenges may increase during adolescence [14]. Moreover, sex-based differences in ASD may be amplified as the onset of menses in autistic females can coincide with significant mood and emotion dysregulation [15]. However, other studies have revealed improvement over adolescence for some aspects of ASD symptom presentation [16], to include better behavior regulation (e.g., [14]).

### Puberty

Puberty explicitly refers to biological maturation resulting in secondary sexual characteristics and accompanying changes in cognitive, emotional, and physiological development (e.g., [1]). While puberty broadly coincides with adolescence, the onset can vary widely based on demographic, biobehavioral and environmental factors [17]. In the United States, the mean pubertal onset for breast development (thelarche) in females ranges from 8.8 to 10.3 years [18] and later for boys with a mean onset of genitalia development between 9.5 and 13.5 years [19, 20]. Interestingly, the age of pubertal onset has been steadily dropping in recent decades for both sexes (e.g., [18, 21–23]), including an average of 3-month decline in age of thelarche per decade in females since 1977 as noted by Eckert-Lind et al. [18]. Similarly, in males, recent reports suggest significantly earlier onset as evidenced by other pubertal markers, such as age of voice change [21]. Differences in pubertal timing are considered risk factors for poor mental health outcomes; specifically, early maturation for girls and late maturation in boys [24]. Critically, advanced pubertal timing has been associated with higher rates of depression (e.g., [25–28]).

Recent research has shown advanced pubertal onset in ASD, especially in females [10, 29], which indicates a greater risk of psychological and social challenges. Moreover, several sex-based differences in autism have been observed suggesting careful consideration for their unique and complex biopsychosocial profiles (e.g., [30–32]).

### Depression trajectories in ASD and TD youth

Cross-sectional and longitudinal studies in ASD have shown significant developmental effects related to adolescence in physiological regulation and stress responsivity [32, 33], which have been further associated with depression [34]. It has also been shown that depression occurs earlier during adolescence for autistic youth based on both parental and self-report [35]. Moreover, both diagnostic (ASD higher) and sex-based (female higher) differences in self-rated symptoms of depression [36] have been observed.

For most TD youth, depressive symptoms typically emerge between age 12 and 14 [7, 8] and the interaction of social, psychological, and biological factors at different times during development sets youth on divergent trajectories [37]. A 2018 systematic review and meta-analysis of longitudinal trajectories of depression symptoms in non-clinical pediatric populations synthesized findings among 41,236 youth across 20 studies [38]. Most youth (56%) followed a low/stable depressive symptom trajectory or moderate/stable trajectory (26%). A subset of the sample (12%) followed high, increasing, or decreasing symptom trajectories. Membership in the ‘high’ or ‘increasing’ trajectories were primarily predicted by female sex. The authors suggest sex differences in depression trajectories may be linked to differences in stress sensitivity/exposure, pubertal timing/response, and availability of social and emotional supports. A recent study by Kwong et al. [39] found that from similar initial levels of depressive symptoms at age 11, females experienced steeper increases in depressive symptoms than males over their adolescent years until around the age of 20, when levels of depressive symptoms plateaued and started to decrease for both males and females. Females on average also had an earlier age of peak velocity, or age at which depression symptoms increased most rapidly, which occurred at 13.5 years, compared to males at 16 years. Both males and females demonstrated highest depression symptoms at age 20.4 and 19.6 years, respectively.

Like TD youth, rates of depression in individuals with ASD appear to increase with age between childhood and adolescence [40, 41]. The trajectory of depressive symptoms in youth with ASD have revealed some mixed findings, but collectively indicate autistic youth show higher rates of depression than TD populations [6]. Some studies showed declining depression symptoms with age especially during the adolescent to adulthood transition (e.g., [42]), while others revealed consistently elevated or increasing depression symptoms in older adolescents and young adults with ASD [5, 43]. It is difficult to draw conclusions about depression symptoms and age for autistic youth, as differences in findings may be attributable to measurement variance (e.g., parent/self-report, broadband or depression-specific measures) and sample characteristics. Factors such as age, sex, and cognitive ability level may impact depressive symptom presentations for autistic youth [40, 44, 45], since symptoms may be more prevalent among individuals with greater cognitive capacity (e.g., [45]) and are more prominent in autistic females compared to males [46].

Three studies to our knowledge have specifically investigated depression among youth with and without ASD within a longitudinal framework. Gotham et al. [43] examined depression trajectories and predictors among youth aged 6–24 with ASD (n = 99) and non-spectrum developmental disabilities (DD; n = 49). ASD diagnosis and increasing age were associated with higher levels of depression symptoms as measured by the Child Behavior Checklist [47] Affective Disorders subscale and the Adult Behavior Checklist [48] Depressive subscale. Males showed more depression symptoms than females at age 13, though depression symptoms steadily increased throughout adolescence for females resulting in no observed sex differences by age 21. As these findings contrast with research among neurotypical populations, it is important to consider limitations associated with the small sample of autistic females (n = 13). McCauley et al. [49] assessed depression symptoms using the Withdrawn/Depressed subscale from the Aberrant Behavior Checklist [47] across five timepoints among 253 youth referred for autism evaluation. They found that 68.04% of youth demonstrated a stable/low depression symptom trajectory throughout adolescence and early adulthood while 31.96% followed a high/fluctuating symptom trajectory, with symptoms increasing from age 9 to age 20 then decreasing until age 25 [49]. The second study by Rai et al. [5] compared depressive symptom trajectories among 6091 youth (age 10–18) with or without ASD and autistic traits across six timepoints using data from the Avon Longitudinal Study of Parents and Children [5]. Youth with ASD or elevated autistic traits showed more depression symptoms at age 10 on the Short Mood and Feelings Questionnaire [50] than those without ASD or autistic traits; autistic youth continued to show elevated and increasing depression symptoms until age 18. Results corroborate findings by Schwartzman and Corbett [35] and suggest depression symptoms may present earlier and more intensely for autistic youth compared to those without autism. Findings from longitudinal studies of depression among autistic and non-autistic samples suggest social communication challenges may be predictive of later depressive symptoms. However, no studies to our knowledge have rigorously compared depression symptom trajectories using a depression-specific measure (i.e., Child Depression Inventory [51]) among well-characterized samples of youth with ASD and TD within a longitudinal framework.

Diverging trajectories of depression symptoms for autistic and TD youth suggest youth with ASD are vulnerable to depression symptoms amid a changing social landscape and the significant physical and psychological changes occurring during puberty. As deviations in pubertal timing/tempo have been identified in youth with ASD [10, 29], there remains a need to explore how pubertal development is related to depression symptom trajectories among autistic and TD youth.

### Present study

Adolescence is a crucial developmental stage marked by physical maturational changes, social-cognitive advances, interpersonal transitions, and social-contextual changes that may leave youth particularly vulnerable to the onset, persistence, and recurrence of depressive symptoms [52]. Although limited research has explored the trajectory of depression symptoms for autistic and non-autistic youth using a rigorous longitudinal approach, the present study seeks to address this gap.

The purpose of this study was to examine the trajectory of mental health outcomes; specifically, depression in autistic and neurotypical youth over a 4-year longitudinal study. Based on limited research showing elevated symptoms with an upward developmental trajectory [5, 6, 35, 40, 41], it was hypothesized that youth with ASD would evidence more elevated symptoms of depression compared to youth with TD, and group trajectories would increase over adolescence (Hyp 1.1) and that female sex would be associated with increased depression symptoms (Hyp 1.2). It was hypothesized that pubertal development (Genital/Breast and Pubic Hair) would also be associated with increased depression symptoms in ASD adolescents (Hyp 1.3 and 1.4). Finally, it was hypothesized the same pattern effects would be mirrored by clinically significant elevated depression symptoms (T-scores > 65 on CDI), with ASD individuals showing a higher probability of clinically significant depression symptoms, increasing over adolescence, and females showing a higher probability of clinically significant depression symptoms (Hyp 2.1–2.4).

## Methods

The research was carried out in accordance with the Code of Ethics of the World Medical Association (Declaration of Helsinki). The Vanderbilt Institutional Review Board approved the study. Prior to inclusion in the study, informed written consent and assent were obtained from all parents and study participants, respectively.

### Participants

Participants in the ASD and TD groups were recruited from a broad community sample in the southern United States covering a 200-mile radius that targeted medical and health-related services, clinics, research registries, regional autism/disability organizations, schools, and social media platforms. Inclusion criteria for both groups required (a) an intelligence quotient (IQ) score ≥ 70 due to task demands in the source longitudinal study and (b) between age 10:0–13.9 years of age at enrollment. For autistic youth, diagnosis was confirmed by a licensed clinician with expertise in autism (see Diagnostic Procedures below). Children were excluded if taking medications that alter the hypothalamic–pituitary–adrenal (HPA) axis (e.g., corticosteroids) or hypothalamic–pituitary–gonadal axis (e.g., growth hormone), or medical condition known to impact pubertal development (e.g., Cushing’s Disease).

Data were collected as part of a 4-year longitudinal study on pubertal development and stress [53]. Year 1 (Y1) enrollment occurred when the children were between 10-years-0-months and 13-years-11-months of age; thus, in Y2 the sample was 11–14 years, Y3 the sample was 12–15 years, and Y4 the sample was 13–16 years. The diagnostic procedures were completed in Y1; however, the physical exam and psychological measures were completed annually.^2^ The sample demographic information is presented in Table 1. Additionally, the number of participants retained each year is shown in Table 1. The overall attrition rate was 33%, with most dropouts between Years 1 and 2 of the study. The rate of attrition was found to be significantly higher for participants with ASD compared to those TD at years 2 and 3 (*p* = 0.009 and *p* = 0.011, respectively), however there was no significant difference in attrition between the groups by year 4 (*p* = 0.069) (see Additional file 13). The notable drop after Y1 for the ASD group often occurred following completion of the eligibility visit after receiving initial diagnostic confirmation. Previous longitudinal reports of the study sample showed no differences in those retained versus non-retained on key demographic variables (see [29]), thus, data was treated as missing at random (i.e. missingness depends on variables included in the analysis). The mixed effects models used for statistical analyses are robust to this type of randomness when the fixed and random effect components of the model are correctly specified, and therefore conclusions of the findings are consistent [54].

**Table 1:** Sample demographics and descriptive statistics by diagnosis

	N	TD	ASD	Overall	Test statistic
		(N = 104)	(N = 140)	(N = 244)	
Sex: Female	244	0.44 46/104	0.26 36/140	0.34 82/244	X^2^(1) = 9.17^2**^
Ethnicity: Hispanic	244	0.05 5/104	0.08 11/140	0.07 16/244	X^2^(1) = 0.91^2^
Age	244	10.58 **11.62** 12.63 11.71 (1.21)	10.50 **11.25** 12.25 11.43 (1.03)	10.58 **11.33** 12.33 11.55 (1.12)	F_1,242_ = 2.71^3^
Race	244				X^2^(3) = 12.06^2**^
White		0.86 89/104	0.81 114/140	0.83 203/244	
African American		0.02 2/104	0.12 17/140	0.08 19/244	
American Indian		0.00 0/104	0.00 0/140	0.00 0/244	
Asian or Pacific Islander		0.00 0/104	0.01 1/140	0.00 1/244	
More than One		0.12 13/104	0.06 8/140	0.09 21/244	
G/B Development Change (Y4–Y1)	140	2.00 **2.00** 3.00 2.38 (0.94)	1.00 **2.00** 3.00 2.05 (1.21)	1.00 **2.00** 3.00 2.20 (1.11)	F_1,138_ = 2.66^3^
Pubic Development Change (Y4–Y1)	140	2.00 **3.00** 3.00 2.43 (1.12)	1.00 **2.00** 3.00 2.22 (1.20)	1.00 **2.00** 3.00 2.31 (1.16)	F_1,138_ = 1.40^3^
CDI Total Problems T Score Year 1	237	44.00 **49.00** 55.17 51.10 (8.63)	49.00 **57.00** 66.00 58.75 (12.45)	46.00 **53.00** 63.00 55.46 (11.58)	F_1,235_ = 26.50^3***^
CDI Total Problems T Score Year 2	182	43.67 **47.00** 57.00 50.55 (9.55)	46.67 **55.00** 63.33 56.29 (12.11)	44.00 **50.00** 62.08 53.48 (11.27)	F_1,180_ = 11.09^3***^
CDI Total Problems T Score Year 3	168	44.00 **52.00** 59.00 53.75 (11.32)	44.67 **54.00** 60.00 54.52 (10.55)	44.00 **52.00** 60.00 54.14 (10.91)	F_1,166_ = 0.66^3^
CDI Total Problems T Score Year 4	159	43.42 **50.00** 59.00 52.24 (10.64)	45.00 **52.00** 59.00 53.99 (11.33)	44.00 **52.00** 59.00 53.15 (11.00)	F_1,157_ = 1.16^3^
CDI Emotional Problems T Score Year 1	237	45.00 **50.00** 55.00 51.06 (8.15)	47.00 **55.00** 62.67 56.67 (10.91)	47.00 **53.00** 58.67 54.25 (10.18)	F_1,235_ = 18.52^3***^
CDI Emotional Problems T Score Year 2	182	43.00 **48.00** 55.67 50.62 (8.98)	45.00 **51.00** 61.00 54.18 (11.29)	44.00 **50.00** 58.00 52.44 (10.36)	F_1,180_ = 4.73^3*^
CDI Emotional Problems T Score Year 3	168	44.00 **50.00** 57.83 52.37 (10.13)	45.00 **50.00** 58.67 52.92 (10.63)	44.00 **50.00** 58.00 52.65 (10.36)	F_1,166_ = 0.07^3^
CDI Emotional Problems T Score Year 4	159	44.42 **49.00** 58.00 52.16 (10.14)	44.00 **50.00** 57.00 52.82 (11.07)	44.00 **50.00** 57.83 52.50 (10.61)	F_1,157_ = 0.05^3^
CDI Functional Problems T Score Year 1	237	42.00 **50.00** 57.00 50.85 (9.25)	48.00 **57.00** 68.83 59.44 (13.57)	45.67 **54.00** 63.00 55.74 (12.62)	F_1,235_ = 27.35^3***^
CDI Functional Problems T Score Year 2	182	42.00 **48.00** 57.00 50.29 (9.68)	47.00 **56.00** 65.33 57.28 (12.91)	44.00 **51.00** 62.00 53.86 (11.94)	F_1,180_ = 14.82^3***^
CDI Functional Problems T Score Year 3	168	44.17 **53.00** 61.83 54.45 (11.88)	47.00 **54.00** 61.33 55.00 (10.20)	47.00 **54.00** 61.58 54.73 (11.03)	F_1,166_ = 0.52^3^
CDI Functional Problems T Score Year 4	159	41.00 **49.00** 57.17 51.76 (11.17)	44.00 **53.00** 62.00 54.24 (11.41)	44.00 **52.00** 59.00 53.06 (11.33)	F_1,157_ = 2.51^3^

Median values are highlighted in bold

N is the number of non-missing value. ^1^Kruskal–Wallis. ^2^Pearson. ^3^Wilcoxon

Q1 **Median** Q3

Mean (SD)

*Y* year, *G/B* genital/breast, *CDI* child depression inventory

*p ≤ 0.05, **p ≤ 0.01, ***p ≤ 0.001

In Y1, the analytical sample included 244 total youth, with 239 participants that completed the physical exam described below. The ASD group consisted of 140 participants (median age 11.2) including 36 females and 104 males. The TD group consisted of 104 participants (median age 11.6) including 46 females and 58 males. Of the 239 participants to complete the physical exam, one ASD male was missing measurement for G stage, and one TD female was missing measurement for PH stage, resulting in 238 non-missing measurements for GB and PH stage at Y1. The racial and ethnic characterization of the sample was comprised of 7.9% Black, 83.3% White, 8.7% multiracial, and less than 1% Asian or Pacific Islander.

### Diagnostic procedures

The diagnosis of ASD was based on the Diagnostic and Statistical Manual-5 [3] and confirmed by a psychologist, psychiatrist, or behavioral pediatrician with autism expertise, current clinical judgment by a study team member, and corroborated by the Autism Diagnostic Observation Schedule (ADOS-2) [55]. In Y1, participants with suspected or confirmed autism diagnosis completed a comprehensive assessment at an initial eligibility visit to be included in the ASD group. Because the ADOS-2 (described below) and other gold-standard measures have not been sex-normed, it has been suggested that established cut-offs differentially and inaccurately exclude females [56]. In the current study, two females in the ASD group had ADOS total scores below the cut-off of 7 (total score = 5); however, they were included in the study based upon clinical judgement from the study PI and licensed clinical psychologist (BAC). Furthermore, an equal number of males and females (N = 2 each) were excluded for not meeting diagnostic criteria, and in all cases, participants were evaluated by the PI and excluded based upon clinical judgement and evaluation. Following the visit, all participants received a research letter containing the diagnostic results from the measures outlined below.

*Autism Diagnostic Observation Schedule-Second Edition* (ADOS-2) [55] is a semi-structured interactive play and interview-based instrument used to support the diagnosis of ASD. The ADOS Module 3 was administered by research-reliable personnel. ADOS-2 Module 3 has good item reliability, with mean exact agreement of 88.2% across items, and good test–retest and interrater reliability (0.87). It also has good structural validity and demonstrates high sensitivity (91%) and specificity (84%).

*Social Communication Questionnaire* (SCQ) [57] is a screening instrument for symptoms of ASD. The SCQ was administered to both ASD and TD children. A score of 15 is suggestive of ASD. The SCQ has shown good diagnostic sensitivity (0.71–0.78) and specificity (0.57–0.71), especially when used in conjunction with the ADOS-2 (sensitivity 0.66–0.73; specificity 0.85–0.92) [58]. The current study utilized the Lifetime version, which examines a child’s entire developmental history. TD children with a score ≥ 10 was exclusionary; however, no TD children met that threshold.

*Wechsler Abbreviated Scale of Intelligence, Second Edition* (WASI-II) [59] is a measure of cognitive ability which was used to obtain an estimate of the youth’s intellectual functioning (IQ ≥ 70 required to participate in the study). Internal consistency in children and adolescents is good to excellent (0.87–0.91) and concurrent validity with original, full-length Wechsler tests is acceptable to excellent (0.71–0.92) [59], including within autistic individuals [60].

After an initial eligibility visit, study participants with confirmed classification as ASD or TD, and meeting inclusion criteria, participated in the study visit at Y1 and annually for Y2, Y3 and Y4.

### Measures

Pubertal development was rigorously measured using three different approaches: a physical exam, a parent-report measure, and self-report based on visual representation of Tanner stages [19, 20]. The current study used physical exam as the primary dependent variable for pubertal development based on findings showing the physical exam to be optimal for precise pubertal measurement and superior to parent- and self-reports [61].

*Physical Examination.* The gold standard physical exam was completed at each annual visit to reliably identify pubertal development and assign Tanner stage [19, 20]. The standardized exam obtained two measures with 5 stages from 1 (not begun) to 5 (fully developed) for Male External Genitalia (G1–G5 for males) and Female Breast (B1–B5 for females) (G/B stage) and Pubic hair (P1–P5 for both sexes) (PH stage). Visual inspection and categorization of pubertal and genital stage was completed without palpation of breasts or measurement of testes, which was consistent with the original Tanner staging and maximized participation.

Exams were conducted by trained, licensed male and female study physicians. At the introduction to the participant, physicians spent roughly 5-min establishing rapport, explain the rationale for the exam and address any concerns. Subsequently, height and weight were obtained. During the pubertal stage exam, the adolescent was requested to loosen clothing to fully expose breast and lower genital region, rather than disrobing, which aided in the level of comfort for the participants. A parent or same-gender research member was offered to accompany the participant in the exam room. Inter-rater reliability was established between study physicians on ten randomly selected participants. Cohen’s Kappa (κ) was calculated between study physicians to assess the degree to which raters were able to identify Tanner stages for G/B and PH markers. Inter-rater reliability for markers ranged from κ = 0.62 to 0.75 (all *p* < 0.001; substantial agreement). Absolute agreement was 0.75. Kappa was also calculated to assess the extent to which physicians were able to reliably and independently identify when participants had initiated pubertal maturation (Stage 2) for each marker. Kappa ranged from 0.62 to 1.00 (good to very good). In cases of disagreement, physician ratings were never greater than one stage difference.

*Child Depression Inventory* (CDI) 2nd Edition (CDI-2) [51], is a 28-item self-report questionnaire for children 7–17 years that measures cognitive, affective, and behavioral symptoms of depression over the past two weeks and includes a severity index on a 3-point scale. Questions are assessed across two domains—Emotional Problems (15 items) and Functional Problems (13 items). Raw scores are converted to T-scores based on sex and age-range (7–12 years and 13–17 years) and categorized in ranges of ≤ 59 *Average*, 50–64 *High-average*, 65–69 *Elevated*, and ≥ 70 *Very Elevated* considered clinically significant. Based on the manual, Total T-scores ≥ 65 indicate clinical depressive symptoms and can be classified as a binary outcome which is consistent with a diagnosis of depression [51]. This dichotomized approach has been used in various studies (e.g., [62, 63]). Despite potential concerns regarding self-report measures, recent research using the CDI supports its use in cognitively able autistic youth [35, 36], with previously published item analyses showing distinct diagnostic- and sex-based differences (see [36] for complete item analysis). Moreover, a recent paper examining suicidality demonstrated that approximately 1 out of 5 autistic youth reported suicidal thoughts on the CDI self-report measure but not on a clinician-administered measure [64]. Internal reliability for the CDI is high in both TD (0.67–0.91) [51] and ASD (0.80) [65] individuals.

### Statistical analysis

*Hyp 1.1:* To examine differences in depressive symptoms between diagnostic groups, we fit a linear mixed effects model with robust standard errors on total CDI t-scores with a random intercept by participant. We allowed the diagnosis effect to vary through development, by including a diagnosis by age interaction, and fitting age nonlinearly via natural cubic splines with two degrees of freedom. The differences between diagnostic groups over adolescent development were tested with the diagnosis by age interaction and was then assessed by plotting predicted total CDI scores by age stratified by diagnosis. Within this model, we adjusted for the following covariates: biological sex, use of psychoactive medication, and if the examination was conducted during a peak COVID year. To investigate biological sex differences in depression symptoms (*Hyp 1.2*), we tested the main effect of sex in this model.

*Hyp 1.3 and 1.4:* We used a similar model to Hyp. 1.1, but instead examined differences between diagnostic groups over pubertal development using genital-breast development stage (*Hyp 1.3*) and pubic development stage (*Hyp 1.4*) in place of age. These models were fit separately for each type of Tanner staging.

*Hyp. 2:* To estimate the degree to which ASD, age, and pubertal development were associated with a higher risk of clinically elevated depressive symptoms, we performed parallel analyses with logistic mixed effects models using the same covariates and random effects structure as in *Hyp 1,* instead with the binary indicator for clinically significant elevated depressive symptoms (T-scores > 65 on CDI) as the response. As before, testing for biological sex differences (Hyp 2.2) in terms of clinically elevated depressive symptoms was done using the main effect of sex in the model containing the diagnosis by age interaction.

Exploratory analyses of the emotional and functional subdomains were performed using the same models described above and are included in the supplementary material. All statistical models were fit in R using lme4 and test statistics were computed using type 2 sum of squares ANOVA. We used a robust effect size index (RESI) [66] to power our analyses because it is robust to model misspecification and widely defined across a range of model types making comparing effect sizes across models easily interpretable.

Additional analyses were conducted to investigate depressive trajectories differed by sex in addition to diagnosis, pubertal timing differences in depressive trajectories, as well as the confounding effect of age and pubertal stage. To test whether depression trajectories differed by sex, we reran models from Hyp 1.1–1.4 with an additional sex by diagnosis by age/pubertal stage interaction. The pubertal timing models were modifications of those in Hyp 1.3 and 1.4, adding and testing the diagnosis by age by pubertal stage interaction. The age by puberty interaction quantifies how the effect of puberty on depression differs by age (i.e. pubertal timing) and the three-way interaction tests whether timing differs by diagnosis. Finally, we assessed the confounding effect of age and pubertal stage by comparing a model with both a diagnosis by age and diagnosis by pubertal stage interaction to their corresponding nested model (Hyp 1.3 or Hyp 1.4) and observing how the effects changed. These analyses were also replicated for the elevated depressive symptom models.

While other models were considered for examining pubertal timing by calculating residuals of pubertal stage regressed onto age and sex (e.g., [28]), the aforementioned linear models were used for ease of interpretation and parsimony. For all models, Type II ANOVA tables of main effects are presented in the manuscript, while model outputs and estimates, organized by hypothesis, are presented in Additional files.

## Results

**Fig. 1:**
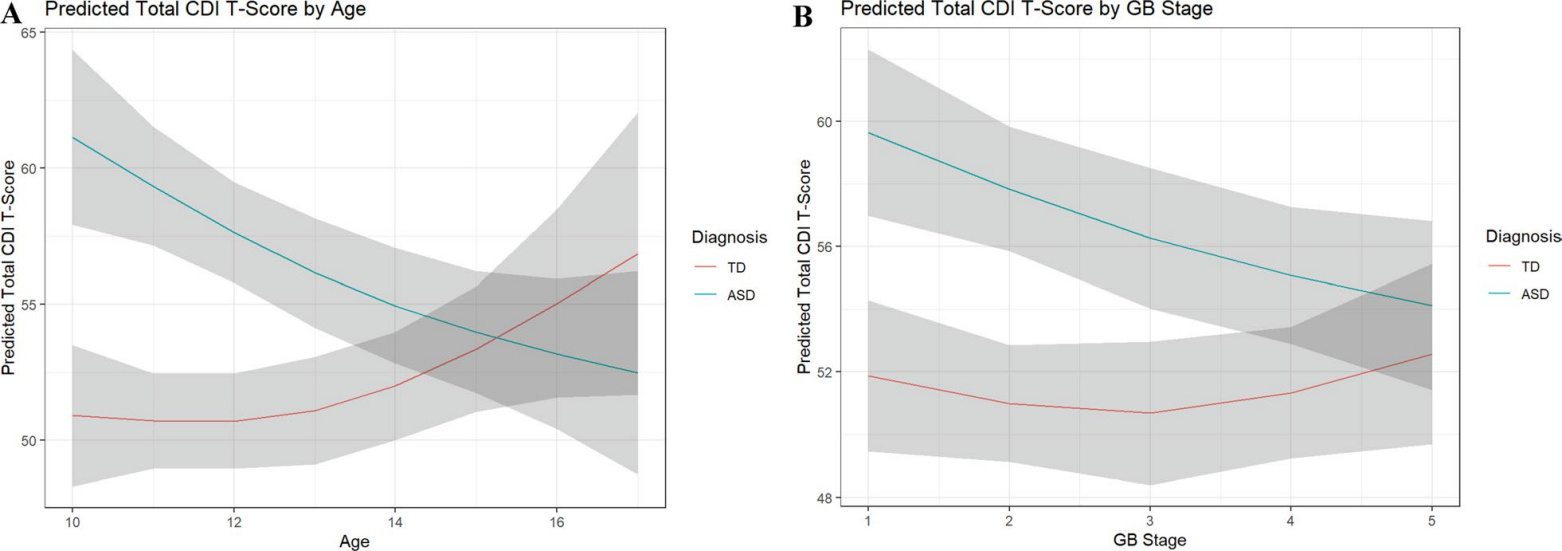
Predicted total CDI T-score by age (**A**) and GB stage (**B**), stratified by diagnosis. *Note:* Shading show’s 95% confidence intervals and overlap suggests no significant difference

*Hyp 1.1–1.2:* We used a linear mixed effect model to investigate the association of ASD, age, and biological sex with increased depression levels. We found a significant diagnosis-age interaction (*p* < 0.001; Table 2): predicted total depressive scores were higher in the ASD group compared to the TD group until approximately 14 years old, and decreased with age, whereas the TD group increased nonlinearly with age starting at approximately 13 years old (Fig. 1A). In addition, females had higher total depressive scores compared to males (*p* = 0.001; Table 2 and Additional file 5).

**Table 2:** Type II ANOVA tables for aim 1: trajectory of depressive symptoms across diagnosis

	Χ^2^	df	*p* Value	Effect size (RESI)
Main effects for age-diagnosis interaction model				
Diagnosis	22.120	1	< 0.001	0.299
Age (nonlinear)	2.468	2	0.291	0.044
Peak COVID year	0.013	1	0.908	0.000
Sex	12.005	1	0.001	0.215
Medication	2.222	1	0.136	0.072
Diagnosis:age (nonlinear)	19.779	2	< 0.001	0.274
Main effects for GB stage-diagnosis interaction model				
Diagnosis	18.750	1	< 0.001	0.276
G/B development stage (nonlinear)	4.582	2	0.101	0.105
Peak COVID Year	0.432	1	0.511	0.000
Sex	13.774	1	< 0.001	0.234
Medication	5.962	1	0.015	0.146
Diagnosis:G/B development stage (nonlinear)	7.078	2	0.029	0.148
Main effects for PH stage-diagnosis interaction model				
Diagnosis	16.999	1	< 0.001	0.261
PH Development stage (nonlinear)	6.978	2	0.031	0.146
Peak COVID year	0.770	1	0.380	0.000
Sex	14.415	1	< 0.001	0.239
Medication	6.009	1	0.014	0.146
Diagnosis:PH development stage (nonlinear)	7.279	2	0.026	0.150

Peak COVID Year defined as 0 = exam not during peak COVID or 1 = exam occurred during peak COVID

*G/B* genital/breast, *PH* pubic hair

*Hyp 1.3 and 1.4:* We fit linear mixed models separately for GB stage and PH stage to investigate associations of depression severity with pubertal stage. As with age, we observed significant interaction effects between diagnosis and GB stage (*p* = 0.029; Table 2 and Additional file 6), as well as diagnosis and PH stage (*p* = 0.026; Table 2 and Additional file 7). Depressive scores on the CDI were elevated in ASD youth in early puberty and decreased with advancing genital/breast stage, whereas a slight increase with advancing stage was observed in TD youth beginning around GB stage 3 (Fig. 1B). Similar developmental trends were observed for PH stage. Overall, total depressive scores appeared to be relatively stable across all pubertal development stages in TD youth for both pubertal development indicators (Fig. 1B and Additional file 1).

**Fig. 2:**
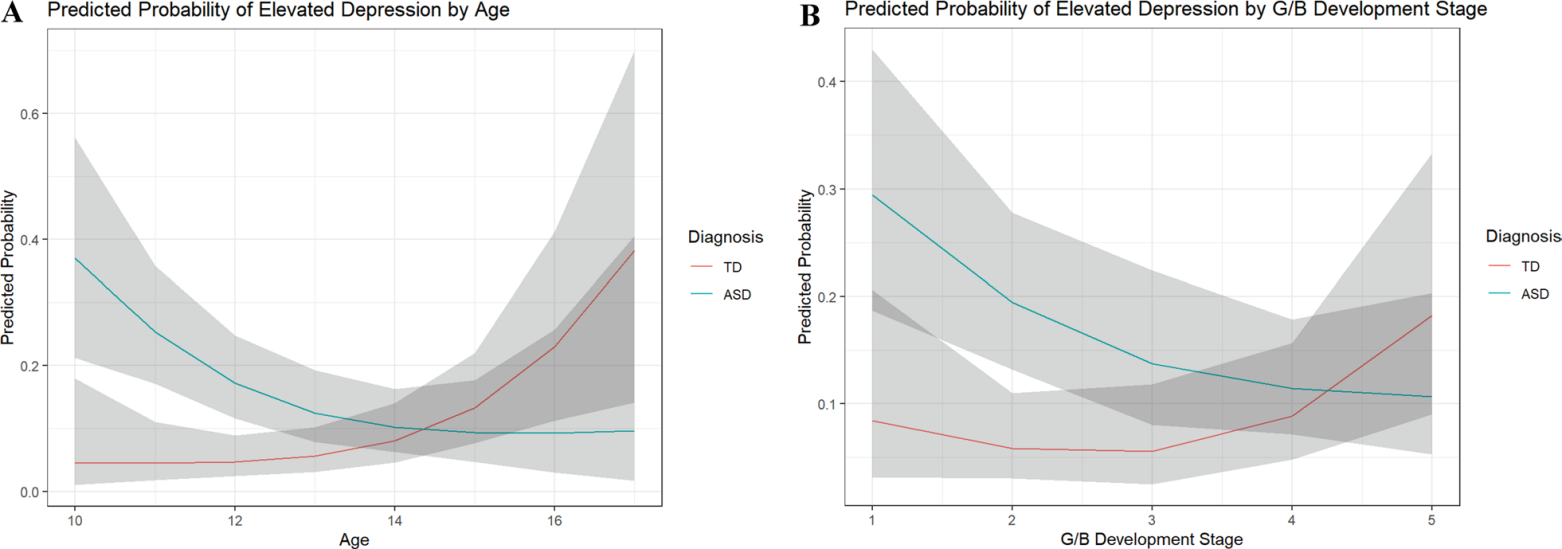
Predicted probability of elevated depression by age (**A**) and GB stage (**B**), stratified by diagnosis. *Note:* Shading show’s 95% confidence intervals and overlap suggests no significant difference

*Hyp 2.1 and 2.2:* In order to study associations with clinically elevated levels of depression, we categorized individuals based on their CDI depression score and modeled it as the response in a logistic mixed effects model. Individuals with ASD showed approximately 37% and 17% risk of experiencing depressive symptoms at ages 10 and 12, respectively, which continued to decrease with age, whereas those in the TD group show the opposite trend beginning with a risk of 5% increasing up to 38% by age 17 (Table 3 and Additional file 8; Fig. 2A). Consistent with results in Hyp 1.2, females had higher odds of experiencing elevated total depressive symptoms while holding all other variables constant (OR = 2.24, *p* = 0.014; Table 3 and Additional file 8).

**Table 3:** Type II ANOVA tables for aim 2: logistic mixed effects for presence of clinically-significant depressive symptoms by diagnosis

	Χ^2^	df	*p* Value	Effect size (RESI)
Main effects for age-diagnosis interaction model				
Diagnosis	3.870	1	0.049	0.110
Age (nonlinear)	10.233	2	0.006	0.186
Peak COVID Year	0.001	1	0.971	0.000
Sex	6.092	1	0.014	0.147
Medication	1.524	1	0.217	0.047
Diagnosis:age (nonlinear)	11.386	2	0.003	0.199
Main effects for GB stage-diagnosis interaction model				
Diagnosis	3.469	1	0.063	0.103
G/B development stage (nonlinear)	5.503	2	0.064	0.123
Peak COVID year	0.267	1	0.606	0.000
Sex	10.963	1	0.001	0.207
Medication	3.876	1	0.049	0.111
Diagnosis:G/B development stage (nonlinear)	8.517	2	0.014	0.167
Main effects for PH stage-diagnosis interaction model				
Diagnosis	3.892	1	0.049	0.111
PH development stage (nonlinear)	6.112	2	0.047	0.133
Peak COVID Year	0.604	1	0.437	0.000
Sex	11.005	1	0.001	0.207
Medication	4.128	1	0.042	0.116
Diagnosis:PH development stage (nonlinear)	5.72	2	0.057	0.126

Peak COVID Year defined as 0 = exam not during peak COVID or 1 = exam occurred during peak COVID

*G/B* genital/breast, *PH* pubic hair

*Hyp 2.3 and 2.4:* Mirroring results in hypothesis 1.3, we observed a significant diagnosis by genital/breast development stage interaction effect on the probability of experiencing elevated depressive symptoms (*p* = 0.014; Table 3 and Additional file 9). Those with ASD had a higher risk of experiencing elevated depressive symptoms (29% at stage 1) which decreased with advancing genital/breast development stage flattening out at stage 4 with 11% risk of elevated depression symptoms. In contrast, the TD group had initially low and stable probabilities (5–8% from stages 1–3) of experiencing elevated depressive symptoms, which increased starting at stage 3 (Fig. 2B) before finally peaking at stage 5 with an elevated depression symptoms risk of 18%. For PH stage, lower pubertal stage was associated with increased risk of clinically significant depression in the ASD group, whereas risk increased in later stages for TD individuals, though the result was not statistically significant (*p* = 0.057; Table 3 and Additional file 2; Additional file 10).

The peak COVID year effect was not found to be significant in any of our models (Tables 2, 3).

The trajectories for the emotional and functional subdomains exhibited similar patterns with the functional subdomain having the stronger effect (Additional files 3 and 4; Additional files 11 and 12).

When examining more complex models, depressive trajectories were not found to differ significantly by sex in addition to diagnosis across age, genital/breast development stage, or pubic hair development stage, respectively (*p* = 0.833, *p* = 0.636, *p* = 0.716). Additionally, pubertal timing was not found to significantly differ between diagnostic groups in either case of using genital/breast development stage or pubic hair development stage as the indicator for puberty (*p* = 0.135, *p* = 0.386). Further, we found that both the genital/breast and pubic hair development stage by diagnosis interactions did not retain significance in the presence of an age by diagnosis interaction. However, the age by diagnosis interaction retained a weak association with total depressive score in the presence of the genital/breast development stage (X^2^ = 4.78, DF = 2, *p* = 0.092, RESI = 0.109) or pubic hair development stage (X^2^ = 5.78, DF = 2, *p* = 0.056, RESI = 0.127) interactions. The corresponding logistic mixed effects models on the elevated depressive score indicators mirrored these results. Overall, these additional analyses support the simpler model structure proposed in hypotheses 1 and 2.

## Discussion

Adolescence is a time of dynamic social, cognitive, and physical maturation that may leave youth, especially with autism, vulnerable to the onset, persistence, and recurrence of depressive symptoms [36, 43, 52]. Surprisingly few studies exist examining the course of mental health outcomes while considering diagnostic (ASD, TD), developmental (age, puberty) and biological sex (male, female) factors that may moderate outcome. The current study investigated the trajectory of depression symptoms in a large well-characterized sample of autistic and non-autistic youth using a rigorous, 4-year longitudinal design. Primary findings revealed significant differences in autistic youth showing higher depression symptoms earlier in development (age, pubertal stage), then decreasing during later development, whereas TD youth show the opposite pattern. Moreover, sex differences were observed in both groups with females showing higher symptoms of depression. While different models were explored examining interaction effects (age, sex, pubertal stage, timing), a simpler model structure based on hypotheses was supported.

It was predicted that autistic youth would evidence more elevated symptoms of depression compared to TD youth, which would increase over adolescence and pubertal development, and these hypotheses were partially confirmed. Specifically, autistic youth had elevated CDI scores compared to TD peers in early adolescence when the sample was between 10 and 14 years of age (Y1 and Y2 of the study), which is consistent with previous research using self-report [36] and parent-report [43] measures. The autistic children evidenced significantly higher symptoms of depression during younger ages than TD youth, corresponding to early adolescence [35]. In one of the few studies comparing ASD and TD groups longitudinally, Rai et al. [5] also showed higher rates of depression symptoms early at 10 years that increased with age.

In contrast to predictions regarding the trajectory, total depressive scores for the ASD group were higher during early adolescence, then decreased with age at the start of middle adolescence rather than continue to rise over development as hypothesized. Conversely, the course of depression scores in the TD group was much lower during early adolescence; yet started to rise around 13 years old. Thus, as the autistic youth reached middle adolescence, depression scores declined while TD youth began to show a steady rise in rates of depressive symptoms. The reported rate of symptoms was most similar between the groups around 14 years of age, but the direction of the trajectory diverged.

The symptom plateau in the autistic adolescents aligns with some studies showing declining depression symptoms with age especially during the adolescent to adulthood transition [42]. However, other studies have reported consistently elevated or rising symptoms of depression in older adolescents and young adults with ASD [5, 43]. Since the current study did not include youth beyond 16 years, it is unclear if the sample would experience higher rates of depressive symptoms as they approach and enter the adult transition. For TD youth, rates were lower early in adolescence but began to rise during the start of middle adolescence. The observed pattern coincides with the often-observed dramatic rise in the onset of psychiatric conditions by middle adolescence in non-autistic youth [2].

Taken together, the rates of depression symptoms and diagnoses in ASD present early and rise with age between the childhood-to-adolescence transition [40, 41] and exhibit higher rates of depression than TD populations [6]. Yet there are mixed findings directly related to the timing and persistence of the trajectory. The decline in reported depressive symptoms in ASD is intriguing and may be related to conditions (e.g., medication, treatment) beyond the scope of the current study. Although the study was conducted during the COVID-19 pandemic, we controlled for self-report measures collected over the months of shutdown in our statistical models via a peak COVID-19 restrictions indicator. This measure is not participant-specific, and a higher resolution COVID-19 effect indicator such as a participant questionnaire inquiring about the impact of COVID-19 on day-to-day functioning might have yielded a more significant COVID effect. Moreover, research explicitly examining stress, anxiety and coping during the pandemic, showed that the ASD compared to the TD group experienced significantly more stress, persistent elevated anxiety and poorer coping strategies compared to the TD group from this sample [67]. Therefore, it seems counterintuitive that depressive symptoms would not follow a similar pattern.

Previous research has attempted to explain discrepant findings in symptom presentation based on factors such as age, sex, and cognitive ability [40, 44, 45]. While we observed differences based on sex (autistic females higher) and age (higher in younger ages), these factors do not explain the drop in symptom profile. More complex analytic models did not show significant differences in trajectories in sex across age or puberty. Some research indicates that psychological and social challenges increase during adolescence for autistic youth [14]; conversely, some areas of functioning, such as behavioral regulation, may improve over time [14, 16]. Therefore, it may be the case that participants in the current sample experienced a waning of symptomology or potential reduction related to treatment or developmental factors that modified affective and/or functional experiences. Another explanation may be related to the attrition in the sample over time such that those most severely affected exited the study prematurely. It will be important for future longitudinal studies to try to replicate this finding as there may be a quiescence in depression that occurs alongside certain biopsychosocial or environmental factors that may inform affective outcomes.

To explicitly consider the contribution of puberty distinct from age, the effects of genital/breast (GB) and pubic hair (PH) development were explored. Like the pattern observed with age, significant diagnostic interaction effects were observed for both GB and PH stage*.* Specifically, the initial elevated depressive scores corresponding with earlier stages of puberty in the ASD group, decreased with pubertal maturation; however, an increase in depressive scores was observed in the TD group with advancing pubertal stage beginning around GB stage 3. Previous research has shown advanced pubertal timing in autistic females; namely, breast development in early stages and breast and PH in mid-pubertal stages [29]. Although development trends were observed for PH stage, they were relatively flat across all stages in TD youth.

In some research (e.g., [28]), pubertal timing is defined as the effect of puberty controlling for the effect of age; thus, exploratory analyses were conducted using this approach. Results showed the pubertal effect was no longer significant, yet the age effect was retained thereby having a stronger effect. The finding intimates that the effect of age and diagnosis on depression is partially mediated by the effect of diagnosis and puberty.

The link to both age and puberty suggests that the onset and course of depression is influenced by a combination of biopsychosocial factors that can contribute to a path of vulnerability as well as resilience [37]. A consistent factor that confers greater risk for depression and is commonly observed in both autistic and non-autistic children and adolescents, is female sex, and this risk tends to build over development [46]. Sex-based variations were evident in the current sample such that females had greater total depressive scores compared to males. This is in line with prior research showing higher rates of depression in autistic females [36, 46] and in non-autistic female youth [40, 44, 45].

Parallel analyses investigated elevated depression symptoms falling in the clinical diagnostic range (T-scores > 65 on CDI) hypothesizing interactions between a diagnosis of ASD and advanced development (adolescence and pubertal), as well as female sex would affect the probability of reporting clinically relevant depressive symptoms. Consistent with prior results, females had significantly higher odds of having elevated total depressive symptoms compared to males. Therefore, symptom profiles showed a similar pattern whether examining scores continuously or when grouping the data into high depression responders. Additionally, those with ASD showed decreasing probability of experiencing elevated depressive symptoms with increasing age, whereas those in the TD group showed the opposite trend. Similarly, those with ASD had decreasing probability of experiencing elevated depressive symptoms with advancing genital-breast development stage, whereas those in the TD group had relatively stable probabilities that began increasing from stage 3. We found a nonsignificant difference between diagnostic groups when analyzing the trajectory of elevated depressive symptoms over pubic-hair development stages. Moreover, the absolute number of participants across the group who evidenced clinically-relevant signs of depression followed a similar pattern. For example, in Y1, 27% of the ASD group endorsed high rates of depressive symptoms compared to only 14% in the TD group. In contrast in Y4, 12% of the ASD group endorsed high rates of depression compared to 15% of the TD group.

### Limitations and future directions

Despite being among the first studies to longitudinally examine depression in a well-characterized sample of autistic and TD youth, there are limitations. The study was conducted in part during the COVID-19 pandemic; therefore, the extent to which the social, psychological, and environmental events may have impacted the depression profiles of participants is unclear and beyond the scope of the study. To this end, youth who completed their assessments during this time were flagged and rudimentarily controlled for in the analyses. As noted, a study conducted during the pandemic with the sample showed that the ASD group had significantly more stress and anxiety compared to the TD group [67] suggesting that depression symptoms would likely follow a similar pattern.

Regarding representation, the inclusion in the main study required an intelligence quotient within the average range; thus, the sample was not fully representative of ASD since nearly a third of autistic individuals have co-occurring intellectual disability [9]. There were also fewer females compared to males in the sample, reflecting the higher prevalence of ASD in males [9]. Even so, the male-to-female ratio (3:1) in the current study was comparable to recent studies showing a higher prevalence and representation of autistic females [11]. Despite targeted recruitment efforts, the study sample was primarily White (i.e., 83%); therefore, the extent to which findings apply to diverse and minoritized populations is unknown. Future studies with greater representation in terms of Race and Ethnicity are warranted. While exploratory analyses showed similar trends for functional versus emotional problems between diagnoses, an in-depth analysis of depressive symptoms across emotional, behavioral, and functional domains was beyond the scope of the study. Thus, future research should pursue greater examination of depressive symptoms across dimensions to elucidate a more nuanced understanding of symptom profiles in ASD and TD over time and across development. Similarly, the study did not include a formal clinical assessment of depressive diagnoses. Although analyses examining a binary outcome of clinically significant/not significant symptoms allow for some examination of clinical risk trajectories over time, such an approach limits statistical power. Nevertheless, the results suggest diagnostic (ASD) differences in risk trajectory and offer a foundation for future research to expand these findings with a well-characterized clinical sample. Finally, depressive symptoms were self-reported by the youth without a gold standard clinical interview to verify diagnostic criteria. Self-reported depression symptoms may differ from impressions obtained through formal evaluation. Findings by Gotham et al. [68] highlight that perceived impairment was more strongly associated with depression symptoms than insight (i.e., alignment between self- and expert ratings). Future studies should further examine concordance between self- and clinician- reports of depression symptoms as greater perceived impairment and rumination are associated with increased self-reported depression symptoms among autistic individuals [68]. Nevertheless, the results offer a window into the perceived emotional and functional symptoms experienced by adolescents over time. Future studies are warranted to address these limitations with larger, more representative samples which will allow more complex analytic models that consider multiple latent depression trajectories [38].

## Conclusions

In summary, autistic youth present with higher rates of depressive symptoms compared to TD youth during the childhood-to-adolescent transition. Yet, approaching middle adolescence and puberty, the symptom trajectory in the autistic youth declines coinciding with a steady increase in the TD youth. Findings suggest a potential period of quiescence in depressive symptomology that may be influenced by biopsychosocial factors impacting affective profiles. Future research is needed to replicate and extend these findings to elucidate the dynamic and atypical emotional and functional depressive profiles in autistic youth.

### Supplementary Information

**Additional file 1: Figure S1.** Predicted Total CDI T-Score by PH Stage.

**Additional file 2: Figure S2.** Predicted Probability of Elevated Depression by PH Development Stage.

**Additional file 3: Figure S3.** Predicted Probability of Elevated Depression on CDI Emotional Subscale by Age, GB, and PH Stage.

**Additional file 4: Figure S4.** Predicted Probability of Elevated Depression on CDI Functional Subscale by Age, GB, and PH Stage.

**Additional file 5: Table S1.** Model Output and Estimates for Hyp 1.1 and 1.2.

**Additional file 6: Table S2.** Model Output and Estimates for Hyp 1.3.

**Additional file 7. Table S3.** Model Output and Estimates for Hyp 1.4.

**Additional file 8. Table S4.** Model Output and Estimates for Hyp 2.1 and 2.2.

**Additional file 9. Table S5.** Model Output and Estimates for Hyp 2.3.

**Additional file 10. Table S6.** Model Output and Estimates for Hyp 2.4.

**Additional file 11: Table S7.** Type II ANOVA Tables for CDI Emotional Subscale Models.

**Additional file 12: Table S8.** Type II ANOVA Tables for CDI Functional Subscale Models.

**Additional file 13: Table S9.** Attrition Rates by Diagnosis.

## Data Availability

Data from this study are shared with the National Database for Autism Research (NDAR) (Collection #2683).

## Abbreviations

ASD: Autism spectrum disorder
CDI: Child depression inventory
GB: Genital breast
HPA: Hypothalamic–pituitary–adrenal
IQ: Intelligence quotient
PH: Pubic hair
SCQ: Social Communication Questionnaire
TD: Typically developing
WASI: Wechsler Abbreviated Scale of Intelligence
Y: Year

## Footnotes

1 At the time of writing this manuscript there is controversy regarding the use of terminology and whether *person-first* language in which the individual (e.g., adolescent) is referenced before the condition (e.g., autism) or whether *identity-first* language (e.g., autistic adolescent) should be used. Because such issues have not been resolved, we have opted to take a mixed terminology approach. Similarly, we will use the terms *autism*, *autism spectrum disorder* and *autistic* interchangeably.

2 2 During Y2 and Y3, some participants were unable to complete the full physical examination due to restrictions on in-person lab visits resulting from the COVID-19 pandemic (Y2 N = 43; Y3 N = 59). Nevertheless, the depression questionnaires were completed, and pubertal development scale was estimated based on parent report.
