# Supplementary figures and images for: Trajectory of depressive symptoms over adolescence in autistic and neurotypical youth
Source: Mol Autism. 2024 May 2;15:18. doi: 10.1186/s13229-024-00600-w (PMC11064411; doi:10.1186/s13229-024-00600-w)

## Slide 1
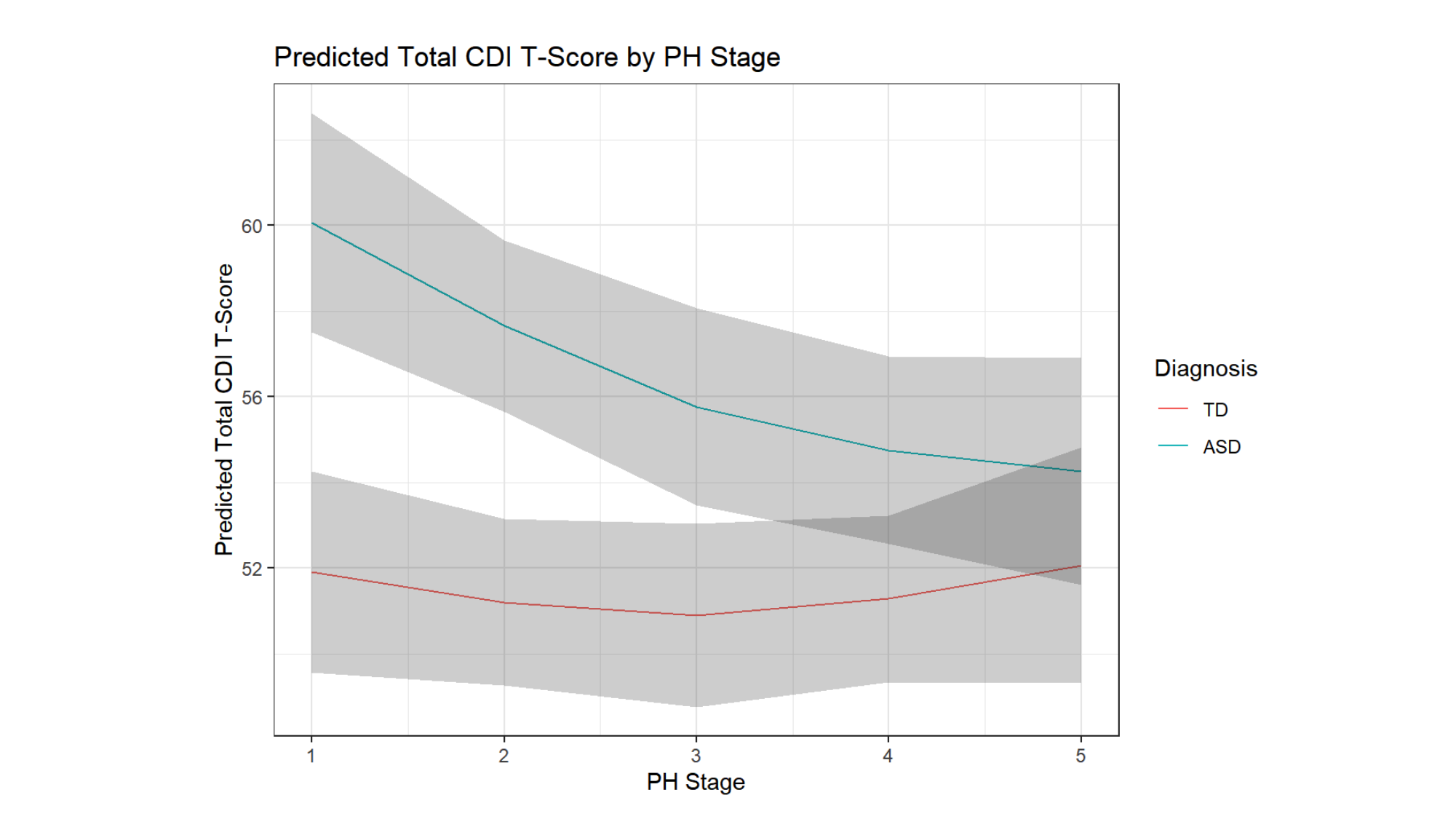

#

Supplement: Supplementary file 1 — Additional file 1: Figure S1. Predicted Total CDI T-Score by PH Stage. [file 13229_2024_600_MOESM1_ESM.pptx]

## Slide 1
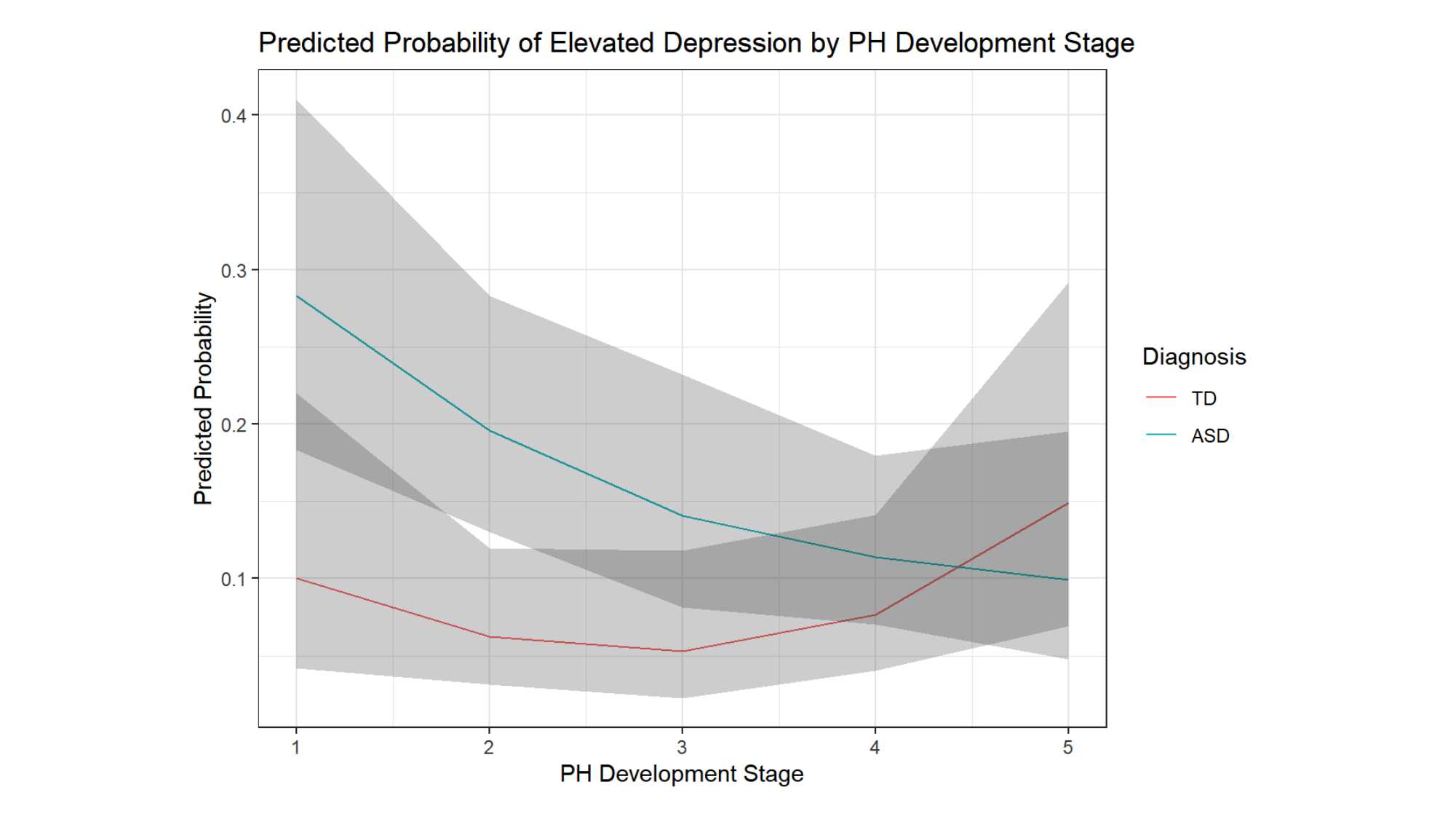

#

Supplement: Supplementary file 2 — Additional file 2: Figure S2. Predicted Probability of Elevated Depression by PH Development Stage. [file 13229_2024_600_MOESM2_ESM.pptx]

## Slide 1
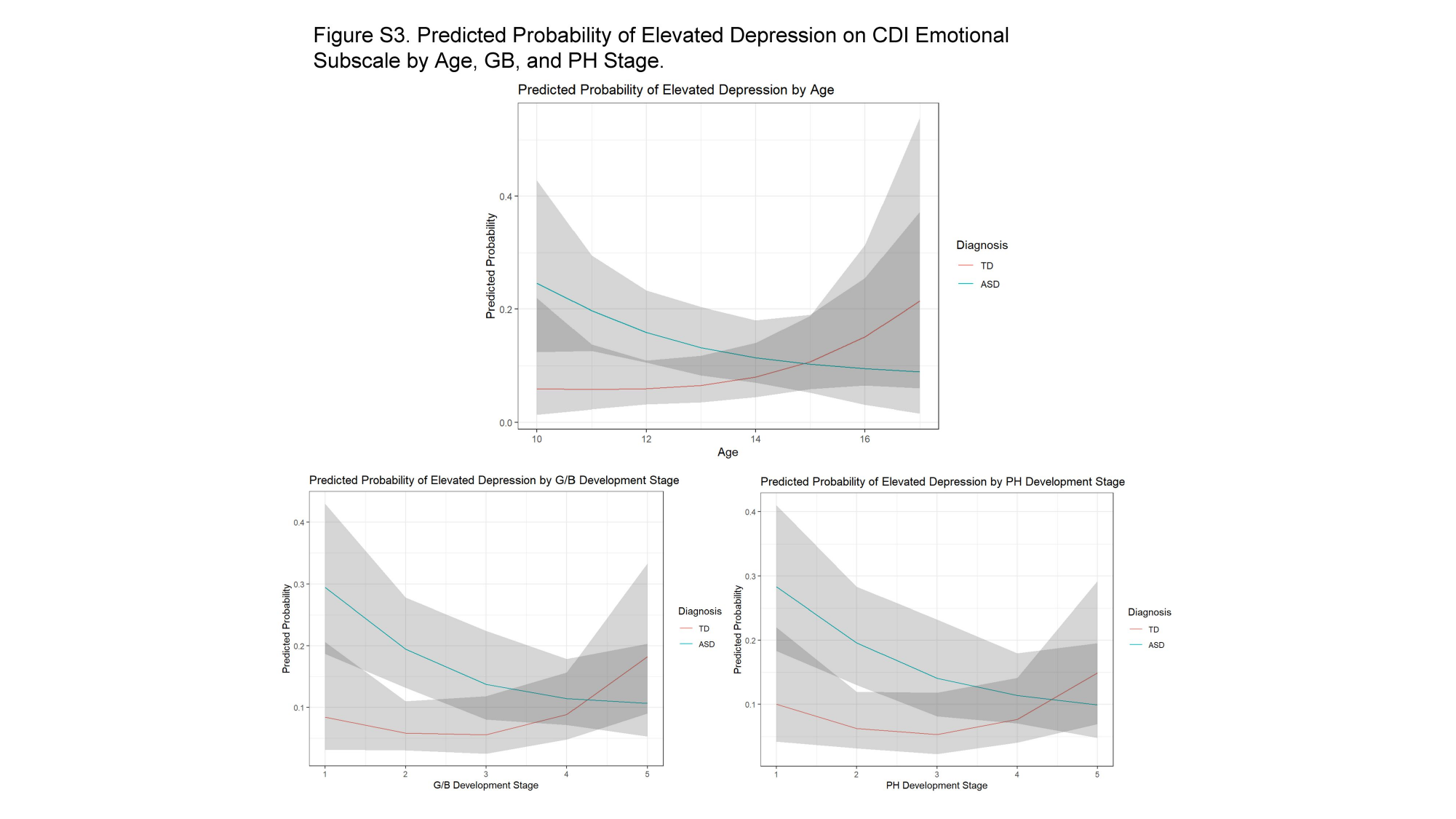

#

Supplement: Supplementary file 3 — Additional file 3: Figure S3. Predicted Probability of Elevated Depression on CDI Emotional Subscale by Age, GB, and PH Stage. [file 13229_2024_600_MOESM3_ESM.pptx]

## Slide 1
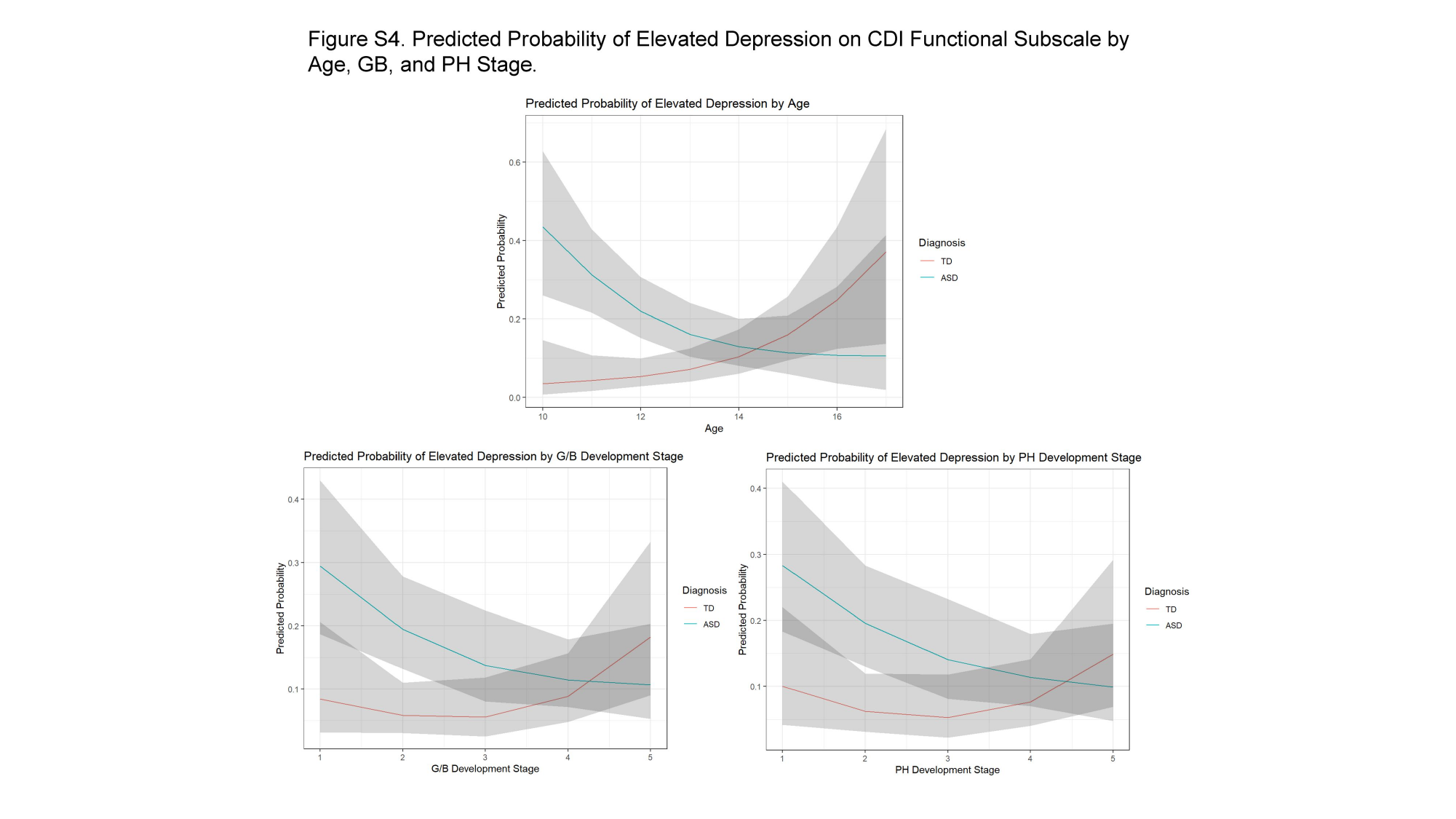

#

Supplement: Supplementary file 4 — Additional file 4: Figure S4. Predicted Probability of Elevated Depression on CDI Functional Subscale by Age, GB, and PH Stage. [file 13229_2024_600_MOESM4_ESM.pptx]
