# Supplementary material for: Trajectory of depressive symptoms over adolescence in autistic and neurotypical youth
Source: Mol Autism. 2024 May 2;15:18. doi: 10.1186/s13229-024-00600-w (PMC11064411; doi:10.1186/s13229-024-00600-w)
Supplement: Supplementary file 5 — Additional file 5: Table S1. Model Output and Estimates for Hyp 1.1 and 1.2. [file 13229_2024_600_MOESM5_ESM.docx]

**Supplemental Table S1. Model Output and Estimates for Hypothesis 1.1 and 1.2**

|  | CDI Total Problems T-Score | | |
| --- | --- | --- | --- |
| **Predictors** | **Estimates** | **95% CI** | **p** |
| (Intercept) | 48.783 | (46.023, 51.542) | <0.001 |
| Diagnosis: ASD | 10.225 | (6.101, 14.349) | <0.001 |
| Age | 3.238 | (-2.603, 9.079) | 0.277 |
| Age' | 6.740 | (0.597, 12.884) | 0.032 |
| COVID Year: Yes | 0.072 | (-1.151, 1.295) | 0.908 |
| Sex: Female | 4.430 | (1.920, 6.941) | 0.001 |
| Medication: Yes | 1.442 | (-0.457, 3.340) | 0.137 |
| Diagnosis:Age | -15.205 | (-24.182, -6.228) | 0.001 |
| Diagnosis:Age' | -12.992 | (-20.408, -5.576) | 0.001 |
| N ID | 237 |  |  |
| Observations | 738 |  |  |
| Random Effects Standard Deviations | | | |
| **Random Effects** | **Standard Deviation** | |  |
| ID | 8.006872 |  |  |
| Residual | 7.274613 |  |  |
| *Note: COVID Year defined as 0 = exam not during peak COVID or 1 = exam occurred during peak COVID.* | | | |
