# Supplementary material for: Trajectory of depressive symptoms over adolescence in autistic and neurotypical youth
Source: Mol Autism. 2024 May 2;15:18. doi: 10.1186/s13229-024-00600-w (PMC11064411; doi:10.1186/s13229-024-00600-w)
Supplement: Supplementary file 6 — Additional file 6: Table S2. Model Output and Estimates for Hyp 1.3. [file 13229_2024_600_MOESM6_ESM.docx]

**Supplemental Table S2. Model Output and Estimates for Hypothesis 1.3**

|  | CDI Total Problems T-Score | | |
| --- | --- | --- | --- |
| **Predictors** | **Estimates** | **95% CI** | **p** |
| (Intercept) | 48.979 | (46.588, 51.370) | <0.001 |
| Diagnosis: ASD | 7.766 | (4.103, 11.429) | <0.001 |
| G/B Stage | -1.521 | (-7.082, 4.040) | 0.591 |
| G/B Stage' | 1.580 | (-1.641, 4.801) | 0.336 |
| COVID Year: Yes | 0.895 | (-1.780, 3.570) | 0.511 |
| Sex: Female | 5.026 | (2.366, 7.686) | <0.001 |
| Medication: Yes | 2.664 | (0.521, 4.807) | 0.015 |
| Diagnosis:G/B Stage | -5.878 | (-13.830, 2.075) | 0.147 |
| Diagnosis:G/B Stage' | -5.441 | (-9.751, -1.131) | 0.013 |
| N ID | 233 |  |  |
| Observations | 605 |  |  |
| Random Effects Standard Deviations | | | |
| **Random Effects** | **Standard Deviation** | |  |
| ID | 7.918965 |  |  |
| Residual | 7.404728 |  |  |
| *Note: COVID Year defined as 0 = exam not during peak COVID or 1 = exam occurred during peak COVID.*  *G/B = Genital/Breast Stage* | | | |
