# Supplementary material for: Trajectory of depressive symptoms over adolescence in autistic and neurotypical youth
Source: Mol Autism. 2024 May 2;15:18. doi: 10.1186/s13229-024-00600-w (PMC11064411; doi:10.1186/s13229-024-00600-w)
Supplement: Supplementary file 7 — Additional file 7. Table S3. Model Output and Estimates for Hyp 1.4. [file 13229_2024_600_MOESM7_ESM.docx]

**Supplemental Table S3. Model Output and Estimates for Hypothesis 1.4**

|  | CDI Total Problems T-Score | | |
| --- | --- | --- | --- |
| Predictors | Estimates | 95% CI | p |
| (Intercept) | 48.901 | (46.609, 51.194) | <0.001 |
| Diagnosis: ASD | 8.158 | (4.619, 11.697) | <0.001 |
| PH Stage | -1.463 | (-5.979, 3.052) | 0.525 |
| PH Stage' | 0.853 | (-2.237, 3.944) | 0.588 |
| COVID Year: Yes | 1.181 | (-1.463, 3.825) | 0.381 |
| Sex: Female | 5.315 | (2.566, 8.064) | <0.001 |
| Medication: Yes | 2.706 | (0.538, 4.873) | 0.015 |
| Diagnosis:PH Stage | -7.457 | (-14.578, -0.335) | 0.040 |
| Diagnosis:PH Stage' | -4.411 | (-8.569, -0.253) | 0.038 |
| N ID | 234 |  |  |
| Observations | 607 |  |  |
| Random Effects Standard Deviations | | | |
| **Random Effects** | **Standard Deviation** | |  |
| ID | 8.189017 |  |  |
| Residual | 7.235330 |  |  |
| *Note: COVID Year defined as 0 = exam not during peak COVID or 1 = exam occurred during peak COVID.*  *PH = Pubic Hair Stage* | | | |
