# Supplementary material for: Trajectory of depressive symptoms over adolescence in autistic and neurotypical youth
Source: Mol Autism. 2024 May 2;15:18. doi: 10.1186/s13229-024-00600-w (PMC11064411; doi:10.1186/s13229-024-00600-w)
Supplement: Supplementary file 8 — Additional file 8. Table S4. Model Output and Estimates for Hyp 2.1 and 2.2. [file 13229_2024_600_MOESM8_ESM.docx]

**Supplemental Table S4. Model Output and Estimates for Hypothesis 2.1 and 2.2**

|  | Elevated Depression (CDI Total > 65) | | |
| --- | --- | --- | --- |
| **Predictors** | **Odds Ratio** | **95% CI** | **p** |
| (Intercept) | 0.031 | (0.007, 0.144) | <0.001 |
| Diagnosis: ASD | 12.269 | (2.236, 67.317) | 0.004 |
| Age | 5.011 | (0.243, 103.480) | 0.297 |
| Age' | 16.401 | (2.969, 90.598) | 0.001 |
| COVID Year: Yes | 1.011 | (0.544, 1.882) | 0.971 |
| Sex: Female | 2.244 | (1.181, 4.264) | 0.014 |
| Medication: Yes | 1.444 | (0.806, 2.587) | 0.217 |
| Diagnosis:Age | 0.010 | (0.000, 0.311) | 0.009 |
| Diagnosis:Age' | 0.025 | (0.002, 0.400) | 0.009 |
| N ID | 237 |  |  |
| Observations | 738 |  |  |
| Random Effects Standard Deviations | | | |
| **Random Effects** | **Standard Deviation** | |  |
| ID | 1.378405 |  |  |
| Residual | 1.813836 |  |  |
| *Note: COVID Year defined as 0 = exam not during peak COVID or 1 = exam occurred during peak COVID.* | | | |
