# Supplementary material for: Trajectory of depressive symptoms over adolescence in autistic and neurotypical youth
Source: Mol Autism. 2024 May 2;15:18. doi: 10.1186/s13229-024-00600-w (PMC11064411; doi:10.1186/s13229-024-00600-w)
Supplement: Supplementary file 9 — Additional file 9. Table S5. Model Output and Estimates for Hyp 2.3. [file 13229_2024_600_MOESM9_ESM.docx]

**Supplemental Table S5. Model Output and Estimates for Hypothesis 2.3**

|  | Elevated Depression (CDI Total > 65) | | |
| --- | --- | --- | --- |
| **Predictors** | **Odds Ratio** | **95% CI** | **p** |
| (Intercept) | 0.049 | 0.017 – 0.144 | <0.001 |
| Diagnosis: ASD | 4.554 | 1.348 – 15.389 | 0.015 |
| G/B Stage | 0.737 | 0.066 – 8.256 | 0.804 |
| G/B Stage' | 3.634 | 1.192 – 11.075 | 0.023 |
| COVID Year: Yes | 1.267 | 0.515 – 3.116 | 0.606 |
| Sex: Female | 2.799 | 1.522 – 5.149 | 0.001 |
| Medication: Yes | 1.802 | 1.003 – 3.238 | 0.049 |
| Diagnosis:G/B Stage | 0.188 | 0.011 – 3.170 | 0.246 |
| Diagnosis:G/B Stage' | 0.132 | 0.031 – 0.559 | 0.006 |
| N ID | 237 |  |  |
| Observations | 738 |  |  |
| Random Effects Standard Deviations | | | |
| **Random Effects** | **Standard Deviation** | |  |
| ID | 1.048809 |  |  |
| Residual | 1.813836 |  |  |
| *Note: COVID Year defined as 0 = exam not during peak COVID or 1 = exam occurred during peak COVID.*  *G/B = Genital/Breast Stage* | | | |
