# Supplementary material for: Trajectory of depressive symptoms over adolescence in autistic and neurotypical youth
Source: Mol Autism. 2024 May 2;15:18. doi: 10.1186/s13229-024-00600-w (PMC11064411; doi:10.1186/s13229-024-00600-w)
Supplement: Supplementary file 10 — Additional file 10. Table S6. Model Output and Estimates for Hyp 2.4. [file 13229_2024_600_MOESM10_ESM.docx]

**Supplemental Table S6. Model Output and Estimates for Hypothesis 2.4**

|  | Elevated Depression (CDI Total > 65) | | |
| --- | --- | --- | --- |
| **Predictors** | **Odds Ratio** | **95% CI** | **p** |
| (Intercept) | 0.058 | 0.022 – 0.149 | <0.001 |
| Diagnosis: ASD | 3.553 | 1.180 – 10.695 | 0.024 |
| PH Stage | 0.424 | 0.046 – 3.875 | 0.447 |
| PH Stage' | 2.639 | 0.805 – 8.650 | 0.109 |
| COVID Year: Yes | 1.431 | 0.579 – 3.536 | 0.437 |
| Sex: Female | 2.946 | 1.556 – 5.578 | 0.001 |
| Medication: Yes | 1.854 | 1.022 – 3.362 | 0.042 |
| Diagnosis:PH Stage | 0.366 | 0.028 – 4.827 | 0.445 |
| Diagnosis:PH Stage' | 0.166 | 0.036 – 0.771 | 0.022 |
| N ID | 237 |  |  |
| Observations | 738 |  |  |
| Random Effects Standard Deviations | | | |
| **Random Effects** | **Standard Deviation** | |  |
| ID | 1.144552 |  |  |
| Residual | 1.813836 |  |  |
| *Note: COVID Year defined as 0 = exam not during peak COVID or 1 = exam occurred during peak COVID.*  *PH = Pubic Hair Stage* | | | |
