# Supplementary material for: Trajectory of depressive symptoms over adolescence in autistic and neurotypical youth
Source: Mol Autism. 2024 May 2;15:18. doi: 10.1186/s13229-024-00600-w (PMC11064411; doi:10.1186/s13229-024-00600-w)
Supplement: Supplementary file 11 — Additional file 11: Table S7. Type II ANOVA Tables for CDI Emotional Subscale Models. [file 13229_2024_600_MOESM11_ESM.docx]

**Supplemental Table S7. Type II ANOVA Tables for CDI Emotional Subscale Models.**

|  | **Χ^2^** | **df** | ***p*-value** | **Effect Size (S)** |  |  | **Χ^2^** | **df** | ***p*-value** | **Effect Size (S)** |
| --- | --- | --- | --- | --- | --- | --- | --- | --- | --- | --- |
| **Hyp 1.1 and 1.2** |  |  |  |  |  | **Hyp 2.1 and 2.2** |  |  |  |  |
| Diagnosis | 13.911 | 1 | <0.001 | 0.233 |  | Diagnosis | 3.176 | 1 | 0.075 | 0.096 |
| Age | 2.435 | 2 | 0.296 | 0.043 |  | Age | 2.484 | 2 | 0.289 | 0.045 |
| Peak COVID Year | 0.140 | 1 | 0.708 | 0.000 |  | Peak COVID Year | 0.004 | 1 | 0.952 | 0.000 |
| Sex | 3.915 | 1 | 0.048 | 0.111 |  | Sex | 1.607 | 1 | 0.205 | 0.051 |
| Medication | 2.303 | 1 | 0.129 | 0.074 |  | Medication | 2.939 | 1 | 0.086 | 0.090 |
| Diagnosis:Age | 14.309 | 2 | 0.001 | 0.228 |  | Diagnosis:Age | 4.288 | 2 | 0.117 | 0.098 |
| **Hyp 1.3** |  |  |  |  |  | **Hyp 2.3** |  |  |  |  |
| Diagnosis | 11.468 | 1 | 0.001 | 0.212 |  | Diagnosis | 2.643 | 1 | 0.104 | 0.084 |
| G/B Stage | 2.388 | 2 | 0.303 | 0.041 |  | G/B Stage | 3.006 | 2 | 0.222 | 0.066 |
| Peak COVID Year | 0.685 | 1 | 0.408 | 0.000 |  | Peak COVID Year | 1.24 | 1 | 0.265 | 0.032 |
| Sex | 4.671 | 1 | 0.031 | 0.126 |  | Sex | 3.406 | 1 | 0.065 | 0.102 |
| Medication | 4.029 | 1 | 0.045 | 0.114 |  | Medication | 4.723 | 1 | 0.030 | 0.126 |
| Diagnosis:G/B Stage | 6.399 | 2 | 0.041 | 0.137 |  | Diagnosis:G/B Stage | 3.723 | 2 | 0.155 | 0.086 |
| **Hyp 1.4** |  |  |  |  |  | **Hyp 2.4** |  |  |  |  |
| Diagnosis | 10.243 | 1 | 0.001 | 0.199 |  | Diagnosis | 2.85 | 1 | 0.091 | 0.089 |
| PH Stage | 4.239 | 2 | 0.120 | 0.098 |  | PH Stage | 3.948 | 2 | 0.139 | 0.091 |
| Peak COVID Year | 1.062 | 1 | 0.303 | 0.016 |  | Peak COVID Year | 1.792 | 1 | 0.181 | 0.058 |
| Sex | 5.247 | 1 | 0.022 | 0.135 |  | Sex | 3.575 | 1 | 0.059 | 0.105 |
| Medication | 4.137 | 1 | 0.042 | 0.116 |  | Medication | 4.897 | 1 | 0.027 | 0.129 |
| Diagnosis:PH Stage | 6.356 | 2 | 0.042 | 0.136 |  | Diagnosis:PH Stage | 2.366 | 2 | 0.306 | 0.040 |
| Note: *Peak COVID Year defined as 0 = exam not during peak COVID or 1 = exam occurred during peak COVID. All Age, G/B Stage, PH Stage, and interaction terms are nonlinear.*  *G/B = Genital/Breast; PH = Pubic Hair* | | | | | | | | | | |
