# Supplementary material for: Trajectory of depressive symptoms over adolescence in autistic and neurotypical youth
Source: Mol Autism. 2024 May 2;15:18. doi: 10.1186/s13229-024-00600-w (PMC11064411; doi:10.1186/s13229-024-00600-w)
Supplement: Supplementary file 12 — Additional file 12: Table S8. Type II ANOVA Tables for CDI Functional Subscale Models. [file 13229_2024_600_MOESM12_ESM.docx]

**Supplemental Table S8. Type II ANOVA Tables for CDI Functional Subscale Models.**

|  | **Χ^2^** | **df** | ***p*-value** | **Effect Size (S)** |  |  | **Χ^2^** | **df** | ***p*-value** | **Effect Size (S)** |
| --- | --- | --- | --- | --- | --- | --- | --- | --- | --- | --- |
| **Hyp 1.1 and 1.2** |  |  |  |  |  | **Hyp 2.1 and 2.2** |  |  |  |  |
| Diagnosis | 21.961 | 1 | <0.001 | 0.297 |  | Diagnosis | 5.035 | 1 | 0.025 | 0.130 |
| Age | 4.135 | 2 | 0.126 | 0.095 |  | Age | 8.154 | 2 | 0.017 | 0.161 |
| Peak COVID Year | 0.082 | 1 | 0.774 | 0.000 |  | Peak COVID Year | 0.903 | 1 | 0.342 | 0.000 |
| Sex | 16.453 | 1 | <0.001 | 0.255 |  | Sex | 9.654 | 1 | 0.002 | 0.191 |
| Medication | 2.695 | 1 | 0.101 | 0.085 |  | Medication | 2.328 | 1 | 0.127 | 0.075 |
| Diagnosis:Age | 19.736 | 2 | <0.001 | 0.274 |  | Diagnosis:Age | 14.616 | 2 | 0.001 | 0.231 |
| **Hyp 1.3** |  |  |  |  |  | **Hyp 2.3** |  |  |  |  |
| Diagnosis | 19.424 | 1 | <0.001 | 0.281 |  | Diagnosis | 4.676 | 1 | 0.031 | 0.126 |
| G/B Stage | 7.458 | 2 | 0.024 | 0.153 |  | G/B Stage | 6.453 | 2 | 0.040 | 0.138 |
| Peak COVID Year | 0.192 | 1 | 0.662 | 0.000 |  | Peak COVID Year | 0.000 | 1 | 0.984 | 0.000 |
| Sex | 18.963 | 1 | <0.001 | 0.278 |  | Sex | 15.645 | 1 | <0.001 | 0.251 |
| Medication | 7.736 | 1 | 0.005 | 0.170 |  | Medication | 6.547 | 1 | 0.011 | 0.154 |
| Diagnosis:G/B Stage | 6.422 | 2 | 0.040 | 0.138 |  | Diagnosis:G/B Stage | 7.871 | 2 | 0.020 | 0.159 |
| **Hyp 1.4** |  |  |  |  |  | **Hyp 2.4** |  |  |  |  |
| Diagnosis | 19.010 | 1 | <0.001 | 0.277 |  | Diagnosis | 4.782 | 1 | 0.029 | 0.127 |
| PH Stage | 9.414 | 2 | 0.009 | 0.178 |  | PH Stage | 8.846 | 2 | 0.012 | 0.171 |
| Peak COVID Year | 0.429 | 1 | 0.513 | 0.000 |  | Peak COVID Year | 0.073 | 1 | 0.787 | 0.000 |
| Sex | 19.412 | 1 | <0.001 | 0.281 |  | Sex | 15.029 | 1 | <0.001 | 0.245 |
| Medication | 7.791 | 1 | 0.005 | 0.170 |  | Medication | 6.746 | 1 | 0.009 | 0.157 |
| Diagnosis:PH Stage | 7.107 | 2 | 0.029 | 0.148 |  | Diagnosis:PH Stage | 6.032 | 2 | 0.049 | 0.131 |
| Note: *Peak COVID Year defined as 0 = exam not during peak COVID or 1 = exam occurred during peak COVID. All Age, G/B Stage, PH Stage, and interaction terms are nonlinear.*  *G/B = Genital/Breast; PH = Pubic Hair* | | | | | | | | | | |
