# Supplementary material for: Trajectory of depressive symptoms over adolescence in autistic and neurotypical youth
Source: Mol Autism. 2024 May 2;15:18. doi: 10.1186/s13229-024-00600-w (PMC11064411; doi:10.1186/s13229-024-00600-w)
Supplement: Supplementary file 13 — Additional file 13: Table S9. Attrition Rates by Diagnosis. [file 13229_2024_600_MOESM13_ESM.docx]

**Supplemental Table 9. Attrition Rates by Diagnosis**

| **Group** | **Y1 Total** | **Y2 Total (Attrition)** | **Y3 Total (Attrition)** | **Y4 Total (Attrition)** |
| --- | --- | --- | --- | --- |
| ASD | 140 | 99 (0.2929) | 90 (0.3571) | 86 (0.3857) |
| TD | 105 | 90 (0.1429) | 84 (0.2000) | 77 (0.2667) |
| *Note: ASD, Autism Spectrum Disorder; TD, Typical Development; Y, Year* | | | | |
